# Gait speed, cognition and falls in people living with mild-to-moderate Alzheimer disease: data from NILVAD

**DOI:** 10.1186/s12877-020-01531-w

**Published:** 2020-03-30

**Authors:** Adam H. Dyer, Brian Lawlor, Sean P. Kennelly, for the NILVAD Study Group, Brian Lawlor, Ricardo Segurado, Sean Kennelly, Marcel G. M. Olde Rikkert, Robert Howard, Florence Pasquier, Anne Bor¨jesson-Hanson, Magda Tsolaki, Ugo Lucca, D. William Molloy, Robert Coen, Matthias W. Riepe, Ja’nos Ka’lma’n, Rose Anne Kenny, Fiona Cregg, Sarah O’Dwyer, Cathal Walsh, Jessica Adams, Rita Banzi, Laetitia Breuilh, Leslie Daly, Suzanne Hendrix, Paul Aisen, Siobhan Gaynor, Ali Sheikhi, Diana G. Taekema, Frans R. Verhey, Raffaello Nemni, Flavio Nobili, Massimo Franceschi, Giovanni Frisoni, Orazio Zanetti, Anastasia Konsta, Orologas Anastasios, Styliani Nenopoulou, Fani Tsolaki-Tagaraki, Magdolna Pakaski, Olivier Dereeper, Olivier Se’ne’chal, Isabelle Lavenu, Gauthier Calais, Fiona Crawford, Michael Mullan, Pauline Aalten, Maria A. Berglund, Jurgen A. Claassen, Rianne A. De Heus, Daan L. K. De Jong, Olivier Godefroy, Siobhan Hutchinson, Aikaterini Ioannou, Michael Jonsson, Annette Kent, Ju¨rgen Kern, Petros Nemtsas, Minoa-Kalliopi Panidou, Laila Abdullah, Daniel Paris, Angelina M. Santoso, Gerrita J. van Spijker, Martha Spiliotou, Georgia Thomoglou, Anders Wallin

**Affiliations:** 1 Department of Age-Related Healthcare, Tallaght University Hospital, Dublin, Ireland; 2 Department of Medical Gerontology, School of Medicine, Trinity College Dublin, Dublin 2, Ireland; 3 Mercer’s Institute for Research on Ageing, St James’s Hospital, Dublin, Ireland

**Keywords:** Dementia, Alzheimer disease, Cognition, Falls

## Abstract

**Background:**

Previous evidence suggests that slower gait speed is longitudinally associated with cognitive impairment, dementia and falls in older adults. Despite this, the longitudinal relationship between gait speed, cognition and falls in those with a diagnosis of dementia remains poorly explored. We sought to assess this longitudinal relationship in a cohort of older adults with mild to-moderate Alzheimer Disease (AD).

**Methods:**

Analysis of data from NILVAD, an 18-month randomised-controlled trial of *Nilvadipine* in mild to moderate AD. We examined: (i) the cross-sectional (baseline) association between slow gait speed and cognitive function, (ii) the relationship between baseline slow gait speed and cognitive function at 18 months (Alzheimer Disease Assessment Scale, Cognitive Subsection: ADAS-Cog), (iii) the relationship between baseline cognitive function and incident slow gait speed at 18 months and finally (iv) the relationship of baseline slow gait speed and incident falls over the study period.

**Results:**

Overall, one-tenth (10.03%, *N* = 37/369) of participants with mild-to-moderate AD met criteria for slow gait speed at baseline and a further 14.09% (*N* = 52/369) developed incident slow gait speed at 18 months. At baseline, there was a significant association between poorer cognition and slow gait speed (OR 1.05, 95% CI 1.01–1.09, *p* = 0.025). Whilst there was no association between baseline slow gait speed and change in ADAS-Cog score at 18 months, a greater cognitive severity at baseline predicted incident slow gait speed over 18 months (OR 1.04, 1.01–1.08, *p* = 0.011). Further, slow gait speed at baseline was associated with a significant risk of incident falls over the study period, which persisted after covariate adjustment (IRR 3.48, 2.05–5.92, *p* < 0.001).

**Conclusions:**

Poorer baseline cognition was associated with both baseline and incident slow gait speed. Slow gait speed was associated with a significantly increased risk of falls over the study period. Our study adds further evidence to the complex relationship between gait and cognition in this vulnerable group and highlights increased falls risk in older adults with AD and slow gait speed.

**Trial registration:**

Secondary analysis of the NILVAD trial (Clincaltrials.gov NCT02017340; EudraCT number 2012–002764-27). First registered: 20/12/2013.

## Background

Gait disorders affect a significant number of community-dwelling older adults and have recently been highlighted as a potential indicator of later cognitive decline. Gait disorders are believed to affect up to one third of older adults aged > 70 years and may be divided based on the particular sensorimotor deficit involved: (i) lower level gait disorders such as osteoarthritis, (ii) mid-level gait disorders which result from a focal neurological lesion such as that seen in Parkinson’s Disease and (iii) higher level gait disorders with no demonstrable deficits in the pyramidal/extrapyramidal/sensory or cerebellar systems (and may be particularly related to cerebrovascular disease) [1–3].

Not only does slow gait speed predict a decline in functional status, but has also been linked with adverse outcomes including mortality in older adults [4–9]. Further, there is now a significant body of evidence linking slow gait speed, and in particular a decline in gait speed, with later risk of cognitive decline and dementia. For instance, in a recently published study from the English Longitudinal Study of Aging, both baseline gait speed and change in gait speed was associated with dementia risk over a follow up of > 10 years [10]. This follows a large body of evidence indicating the strong relationship between slow gait speed and later cognitive decline [11–16].

Whilst the evidence for the relationship between slow gait speed and later dementia risk is mounting, the relationship between gait speed and cognitive trajectories in those with Alzheimer Disease (AD) is less clear. Whilst a handful of studies have evaluated the association between gait speed and dementia in cross-section [17–19], longitudinal analyses are limited. A notable study has demonstrated a decline in gait speed associated with clinical progression in those with Mild Cognitive Impairment (MCI) [20]. In the only longitudinal study of gait speed in those with a diagnosis of dementia, baseline gait-speed was associated with a decline in verbal fluency over a 1 year period, but not in overall cognition [21]. Thus, whilst the cross-sectional relationship between gait and cognition in dementia has been demonstrated, the longitudinal relationship between slow gait speed and cognitive function in AD remains unclear.

There is a significant body of evidence reporting that older adults with a diagnosis of AD are at greater risk of falls than their age matched counterparts without dementia [22–26]. The increased risk represents the interaction of several risk factors such as autonomic symptoms, physical activity profile, orthostatic hypotension and polypharmacy [24]. Several studies have reported associations between spatiotemporal gait parameters and falls risk in older adults with dementia [27], however there are also conflicting results. Many studies have taken place in nursing home or long term care facilities and the association has been less-well explored in community dwelling older adults with a diagnosis of AD, particularly those with mild-to-moderate AD [27]. Further, most studies on community dwelling older adults with AD have either occurred in cross-section or retrospectively with < 12 months follow up [27].

Based on the above evidence and the known adverse effects experienced by those with slow gait speed, it follows that slow gait speed may be an indicator of which patients with AD are particularly vulnerable of further cognitive decline and adverse outcomes such as falls. In the current study, we aimed to assess the relationships between (i) baseline cognition and baseline slow gait speed in mild-moderate AD, (ii) baseline slow gait speed and cognitive decline at 18 months (iii) baseline cognitive impairment and incident slow gait speed at 18 months (decline in gait speed) and (iv) the longitudinal relationship between baseline slow gait speed and incident falls over 18 months in older adults with a diagnosis of mild-to moderate AD.

## Methods

### Setting

The current study was embedded within NILVAD, a randomised controlled trial of the antihypertensive *Nilvadipine* in mild-to-moderate Alzheimer Disease (AD). The trial’s primary outcome of a therapeutic effect of *Nilvadipine* in mild/ moderate AD was not achieved. Briefly, participants were recruited from 23 centres in 9 European countries (Clincaltrials.gov NCT02017340; EudraCT number 2012–002764-27). The full trial protocol, the sub-study protocol in addition to the main trial results have been published elsewhere [28–30]. Ethical approval for the trial was granted from the appropriate National Competent Authorities, Independent Ethics Committees and Institutional Review Boards for all study sites.

Participants once enrolled were randomized to either *Nilvadapine* 8 mg or placebo for the 18 month duration of the study.

### Inclusion/exclusion criteria

The inclusion/exclusion criteria for the NILVAD trial have been previously published [28]. Of note, included participants were those aged > 50 years who met the National Institute of Neurological and Communicative Disorders and Stroke/Alzheimer’s Disease Criteria: NINCDS-ADRDA for AD. Criteria for inclusion was a diagnosis of mild-to-moderate AD, defined as a Mini-Mental State (MMSE) Score between 12 and 26 at trial enrolment and a diagnosis of AD as per the NINCDS-ADRDA criteria. Further, those included had a Mini-Mental State (MMSE) Score between 12 and 26 at trial enrolment, meeting criteria for mild to moderate AD.

Relevant exclusion criteria to the current analysis include a comorbid neurological condition such as Parkinson’s Disease or Huntington’s Disease. Similarly, participants with a history of significant head trauma, known structural brain abnormalities or any other condition known to interfere with cognitive function were excluded. Patients meeting criteria for a substance use disorder were also excluded as were participants with significant cardiovascular disease. For full exclusion criteria, readers are directed to the initial trial protocol.

### Gait measurement

Gait speed was measured as part of the NILVAD frailty sub-study, the protocol of which has been previously published [28]. Briefly, gait speed was measured both at baseline and 18 months. Four markings were placed on an even floor with adhesive tape over a distance of 6 m total. The markings were spaced as follows (i) first mark at start, (ii) second mark at 1 m, (iii) third mark 4 m from mark 2 and (iv) fourth mark 1 m from mark 3. Participants were asked to stand at mark 1 and then instructed to walk from mark 1 to mark 4 at normal walking speed. The stopwatch was started once the participant’s first foot touched/crossed mark two and was stopped once the participant’s last foot crossed mark 3. A stopwatch accurate to two decimal places was used. Thus the total speed represented the time for the participant to walk 4 m. If the total time was > 6 s to complete this 4 m walk (< 0.67 ms^− 1^), gait speed was recorded as slow gait speed. This pre-specified binary cut-off was applied as per previous literature and included in the initial sub-study protocol [23, 28].

Baseline slow gait speed was considered as those below this cut-off at trial initiation and incident slow gait speed defined as those below this cut off at 18 months who did not meet the slow great criteria at baseline.

### Cognitive function and dementia severity assessment

The Alzheimer’s Disease Assessment Scale, Cognitive Subsection (ADAS-Cog) was used to assess cognition and was the primary cognitive endpoint in the NILVAD trial. For the current study, we considered ADAS-Cog at initial visit to be baseline ADAS-Cog and analysed the change in ADAS-Cog at 18 months as the main cognitive outcome. ADAS-Cog sub-scores analysed included the following: word recall task, naming, commands, constructional praxis, delated word recall, ideational praxis, orientation, word recognition task, spoken language ability, comprehension, word finding difficult in spontaneous speech, remembering test instructions.

Dementia severity at baseline and at 18-months was assessed using the Clinical Dementia Rating scale sum of boxes (CDR-Sb) score.

### Medical history and regular medications

A comprehensive medical history was taken from participants/carers at time of study enrolment and updated on follow up visits. A list of concomitant medications was also obtained at time of study enrolment and reviewed at follow up visits in order to identify any changes in concomitant medication usage. Medications were classified according to Anatomic Therapeutic Classification (ATC) codes to ensure consistency. For the current analysis, only medications taken for the entire 18-month study duration were considered. Short term and historical medication use were excluded.

### Statistical analysis

STATA V15.0 (Stata Corp, College Station, Texas, USA) was used for all analysis in the current study, with the significance level considered as *p* < 0.05. Baseline descriptive statistics are reported as means with standard deviations where parametric and medians with Interquartile Ranges (IQR) where appropriate. Univariate analysis of between group differences employed T-tests, Wilcoxon rank-sum tests and Chi-square tests. Logistic regression was used to assess predictors of slow gait speed at baseline and adjusted Odds Ratios (OR), 95% Confidence Intervals (CI) and *p*-values presented as appropriate. Change in ADAS-Cog Scores was considered the difference between the baseline ADAS-Cog score and ADAS-Cog score at 18 months, with an increasing score indicating greater cognitive decline.

In the current analysis, we sought to assess the longitudinal relationship between slow gait speed and cognitive function in older adults with mild-to-moderate Alzheimer Disease. Our research questions were as follows: (i) is there an association at baseline between slow gait speed and baseline cognitive function?, (ii) does slow gait speed at baseline predict greater cognitive decline at 18 months in mild-to-moderate AD?, (iii) does cognitive function at baseline predict the development of slow gait speed at 18 months (incident slow gait speed)?, and finally, (iv) does slow gait speed predict falls in those with AD over an 18-month period.

To assess the cross-sectional relationship at baseline between cognitive function (independent variable, linear) and slow gait speed (dependent variable; binary), we used multivariate logistic regression adjusting for important confounders known to influence gait speed and/or cognition. We adjusted for age, gender, Body Mass Index, study group (Nilvadipine vs.placebo), diagnosis duration, symptom duration, years of formal education, total number of medications, antidepressant and benzodiazepine use, total medical comorbidities, diabetes, hypertension, arthritis, baseline ADAS-Cog and baseline CDR-Sb.

In order to assess whether baseline slow gait speed (independent variable; categorical) was associated with cognitive outcomes at 18 months (dependent variable; linear), mixed effects linear regression was used with country as a random effect. The association was tested unadjusted in the first instance (model 1). The association was then tested controlling for important demographic and AD-related covariates including baseline cognitive function (ADAS-Cog Score), age, gender, study arm, body mass index, years of formal education and diagnosis duration (model 2). Finally we incorporated total number of medical comorbidities and total number of medications (model 3).

For assessment of whether baseline cognition (independent variable; linear) was associated with incident slow gait speed (dependent variable; categorical), logistic regression was used Again the association was tested unadjusted followed by adjustment for baseline ADAS-Cog, age, gender, study arm, body mass index, years of formal education, diagnosis duration (model 2) followed by robust adjustment (model 3) for total number of medical conditions, diabetes mellitus, arthritis, total number of medications, antidepressant use and benzodiazepine use based on known impact on gait speed.

Finally, in order to assess the relationship between slow gait speed (independent variable; categorical) and falls (dependent variable; count), a Poisson regression was used, again unadjusted in the first instance, followed by adjustment for baseline ADAS-Cog, age, gender, study arm, body mass index, years of formal education, diagnosis duration (model 2) in addition to total medical comorbidities and total number of medications (model 3).

Analyses were then repeated by ADAS-Cog sub-scores for each task, with the number of errors on each task analysed by Poisson regression using the same covariates the above models. Finally, we repeated the above models using the CDR-Sb as the dependent variable in order to assess the relationship between slow gait speed and dementia severity at baseline at 18 months.

In all above analyses, the following data were categorical: gender, group (Nilvadipine vs. placebo), slow gait speed (present vs absent), antidepressant use (user vs non-user), benzodiazepine use (user vs non-user), diabetes (present vs absent), arthritis (present vs absent), hypertension (present vs absent). All other variables were continuous as detailed above.

## Results

### Participant characteristics

Of those randomized to either *Nilvadipine* or placebo at baseline (*N* = 511), 465 participants had a gait speed measurement performed. Of these, 369 had full clinical and 18-month follow up data obtained including repeat gait speed and cognitive assessment at 18 months. This population constituted those included in the current analysis. Mean age of included participants was 72.8 ± 8.06 and 63.14% (*N* = 233/369) were female. Overall, 50.68% (*N* = 187/369) were assigned to the treatment group. Median duration of AD symptoms was 3.65 years (IQR: 2.26–5.18) and duration since diagnosis was 0.91 years (IQR: 0.42–2.12). At baseline, mean ADAS-Cog was 32.97 ± 9.81 and mean CDR-Sb was 4.82 ± 2.52.

### Baseline association between slow gait speed and cognition

At baseline, one-tenth (10.03%, *N* = 37/369) of participants had slow gait speed defined as described above. On univariate analysis, those with slow gait speed were older, had a higher BMI, fewer years of education, greater number of total medications and comorbidities, poorer baseline cognition rated using the ADAS-Cog and greater dementia severity rated using the CDR-Sb (all *p* < 0.05, see Table 1). On multivariate analysis controlling for all covariates, increasing age (OR 1.10, 1.04–1.17, *p* < 0.001) and poorer baseline cognition on the ADAS-Cog (OR 1.05, 1.01–1.09, *p* = 0.025) were associated with greater risk of baseline slow gait speed (See Table 2). Similarly, there was a non-significant statistical trend for greater dementia severity using the CDR-Sb as a predictor of slow gait speed under this model (OR 1.19, 0.98–1.45, *p* = 0.08). On analysis of ADAS-Cog subset-scores, there were no specific associations between gait speed and separate cognitive domain subset-scores at 18 months.

**Table 1:** Baseline Differences between Those with Mild to Moderate AD with Slow Gait Speed at Baseline and Those Without (Univariate Analysis). ADAS-Cog = Alzheimer Disease Assessment Scale – Cognitive Subsection; CDR-Sb: Clinical Dementia Rating, Sum of Boxes, BMI: Body Mass Index

Characteristic	No Slow Gait Speed (*N* = 332)	Slow Gait Speed (*N* = 37)	*P*-Value
Age, mean (SD)	72.25 (8.02)	77.68 (6.75)	< 0.001*
Gender, female (%)	207 (62.35%)	26 (70.27%)	0.343
BMI, mean (SD)	25.44 (4.23)	27.97 (5.56)	0.005*
Group, nilvadipine (%)	169 (50.9%)	18 (48.65%)	0.795
Diagnosis Duration, median (IQR)	0.90 (0.43–2.22)	1.01 (0.48–1.71)	0.687
Symptom Duration, median (IQR)	3.69 (2.20–5.25)	3.45 (2.50–4.28)	0.689
Education, median (IQR)	16 (14–18)	14 (12–16)	0.003*
Total Medications, median (IQR)	5 (3–6.5)	5 (4–8)	0.022*
Antidepressant Use, N (%)	126 (37.95%)	18 (48.65%)	0.206
Benzodiazepine Use, N (%)	40 (12.05%)	3 (8.11%)	0.479
Total Comorbidities, median (IQR)	4 (2–5)	4 (3–6)	0.009*
Diabetes, N (%)	22 (6.63%)	5 (13.51%)	0.297
Hypertension, N (%)	94 (28.40%)	14 (37.84%)	0.232
Arthritis, N (%)	26 (7.83%)	4 (10.81)	0.529
Baseline ADAS-Cog, mean (SD)	32.49 (9.49)	37.29 (11.56)	0.002*
Baseline CDR-Sb, mean (SD)	4.67 (2.44)	6.13 (2.84)	0.004*

**Table 2:** Multivariate Analysis of Slow Gait Speed at Baseline. At baseline, increasing age and poorer scores on the ADAS-Cog at baseline was associated with significantly greater likelihood of slow gait speed (dependent variable) at baseline. ADAS-Cog = Alzheimer Disease Assessment Scale – Cognitive Subsection; CDR-Sb: Clinical Dementia Rating, Sum of Boxes, BMI: Body Mass Index

Characteristic	OR (95% CI)	*P*-Value
Age	1.10 (1.04–1.17)	< 0.001*
Gender	1.14 (0.47–2.75)	0.773
BMI	1.08 (0.99–1.16)	0.078
Group	1.00 (0.45–2.21)	0.996
Diagnosis Duration	0.88 (0.64–1.20)	0.417
Symptom Duration	0.94 (0.78–1.14)	0.541
Education (Years)	0.93 (0.83–1.04)	0.214
Total Medications	1.07 (0.91–1.25)	0.421
Antidepressant Use	1.53 (0.69–3.41)	0.298
Benzodiazepine Use	0.36 (0.07–1.60)	0.178
Total Comorbidities	1.10 (0.93–1.31)	0.270
Diabetes	0.96 (0.30–3.11)	0.951
Hypertension	1.76 (0.77–3.98)	0.179
Arthritis	0.86 (0.23–3.20)	0.820
Baseline ADAS-Cog	1.05 (1.01–1.09)	0.025*
Baseline CDR-Sb	1.19 (0.98–1.45)	0.081

### Change in ADAS-cog scores over 18 months

Overall, the mean ADAS-Cog in the included cohort increased by 8.87 (± 9.18), indicating both a clinically significant cognitive decline [31] and a statistically significant progression (t = − 18.56, *p* < 0.001).

### Baseline slow gait speed and cognitive decline at 18 months

Unadjusted, there was no association between baseline slow gait speed and ADAS-Cog scores at 18 months (*β:* − 1.49, − 4.61 – 1.63, *p* = 0.35). There was no association on controlling for baseline ADAS-Cog, age, gender, study arm, BMI, education and diagnosis duration (*β*: − 0.63, − 3.69 – 2.42, *p* = 0.69). Further, following further adjustment for important medical comorbidities and concomitant medication use, there was no association between baseline gait speed and ADAS-Cog scores at 18 months (*β:* − 0.80, − 3.86 – 2.25, p = 0.69) (Table 3). Under the same models, there was no association between slow gait speed at baseline and CDR-Sb score at 18 months either unadjusted (*β:* 0.23, − 0.79 – 1.24, *p* = 0.66), or under model 2 (*β:* 0.11, − 0.92 – 1.14, *p* = 0.84) or model 3 (*β:* 0.16, − 0.92 – 1.15, *p* = 0.83).

**Table 3:** Analysis of The Relationship Between Baseline Slow Gait Speed and Cognition at 18 Months (ADAS-Cog) in Mild to Moderate AD. Under unadjusted or adjusted models, there was no association between baseline slow gait speed and cognition at 18 months (dependent variable). ADAS-Cog = Alzheimer Disease Assessment Scale – Cognitive Subsection

Predictor	Model 1		Model 2		Model 3	
	***β*-Coef (95% CI)**	***P***	***β*-Coef (95% CI)**	***P***	***β*Coef (95% CI)**	***P***
Baseline Slow Gait Speed	−1.49 (−4.61, 1.63)	0.35	−0.63 (−3.69, 2.42)	0.69	− 0.80 (−3.86, 2.25)	0.606
Baseline ADAS-Cog			0.22 (0.13, 0.31)	< 0.001*	0.22 (0.13, 0.32)	< 0.001*
Age			−0.21 (−0.32, − 0.10)	< 0.001*	− 0.22 (− 0.34, − 0.10)	< 0.001*
Gender (Female)			0.94 (−2.80, 0.92)	0.321	0.97 (−2.83, 0.90)	0.310
Study Arm			−0.73 (− 2.49, 1.02)	0.414	− 0.70 (− 2.46, 1.06)	0.437
Body Mass Index			−0.25 (− 0.46 - -0.04)	0.018*	− 0.27 (− 0.48 - -0.06)	0.011*
Education (Years)			0.13 (−0.11–0.37)	0.291	0.14 (− 0.10–0.38)	0.244
Diagnosis Duration			− 0.67 (−1.22 – − 0.12)	0.017	−0.68 (− 1.22 – − 0.12)	0.015
Total Medications					0.17 (−0.18–0.52)	0.345
Total Comorbidities					0.08 (−0.33–0.49)	0.697

### Baseline cognition and incident slow gait speed at 18 months

Overall, 14.09% (*N* = 52/369) of participants with mild to moderate AD met the criteria for incident slow gait speed at 18 month follow up (having had normal gait speed at baseline). Overall, those participants meeting criteria for incident slow gait speed had higher baseline ADAS-Cog scores, however this was not a statistically significant or clinically meaningful difference (36.47 +/− 9.44 vs 36.08 +/− 11.42, t = − 0.23, *p* = 0.82) [31]. Unadjusted, ADAS-Cog score at baseline was associated with a greater likelihood of incident slow gait speed at 18 months (OR: 1.04, 1.01–1.07, *p* = 0.015). This association persisted after controlling for age, gender, BMI, study group, years of education and diagnosis duration (OR: 1.04, 1.01–1.08, *p* = 0.009). Further, after robust covariate adjustment for total medical comorbidities, diabetes, arthritis, total number of medications, benzodiazepines and antidepressant use, the association remained significant (OR: 1.04, 1.01–1.08, *p* = 0.011) (see Table 4). Further, dementia severity at baseline rated using the CDR-Sb was significant under the same models with associations seen unadjusted (OR: 1.17, 1.05–1.30, *p* = 0.003) in addition to under models 2 (OR: 1.16, 1.03–1.30, *p* = 0.018) and 3 (OR: 1.15, 1.01–1.30, *p* = 0.031).

**Table 4:** Association between Baseline Cognition (ADAS-Cog) and incident Slow Gait Speed at 18 Months in Mild to Moderate AD. Under all models, baseline cognition was significantly associated with incident slow gait speed (dependent). ADAS-Cog = Alzheimer Disease Assessment Scale – Cognitive Subsection

Predictor	Model 1		Model 2		Model 3	
	**OR (95% CI)**	***P***	**OR (95% CI)**	***P***	**OR (95% CI)**	***P***
Baseline ADAS-Cog	1.04 (1.01, 1.07)	0.015*	1.04 (1.01–1.08)	0.009*	1.04 (1.01–1.08)	0.011*
Age			1.10 (1.05–1.15)	< 0.001*	1.10 (1.05–1.16)	< 0.001*
Gender (Female)			0.61 (0.32–1.18)	0.143	0.63 (0.32–1.24)	0.178
Study Arm			0.82 (0.44–1.54)	0.534	0.70 (0.37–1.36)	0.294
Body Mass Index			1.09 (1.01, 1.16)	0.023*	1.10 (1.02, 1.18)	0.014*
Education (Years)			1.08 (0.99–1.16)	0.076	1.08 (0.99–1.17)	0.064
Diagnosis Duration			1.07 (0.90–1.26)	0.443	1.07 (0.90–1.26)	0.466
Total Comorbidities					0.96 (0.82–1.11)	0.531
Diabetes Mellitus					0.41 (0.11–1.59)	0.195
Arthritis					0.54 (0.14–2.10)	0.373
Total Medications					1.06 (0.94–1.21)	0.330
Benzodiazepine Use					1.49 (0.55–4.01)	0.433
Antidepressant Use					0.99 (0.49–1.98)	0.989

Cognitive Scores (ADAS-Cog) of those at baseline with baseline slow gait speed and normal gait speed in addition to those meeting criteria for incident slow gait speed are detailed below in Table 5.

**Table 5:** Cognitive Scores (ADAS-Cog) at baseline and 18 months by Gait Speed Group at baseline and 18 Months in Mild to Moderate AD. Data presented as means with standard deviations. ADAS-Cog = Alzheimer Disease Assessment Scale – Cognitive Subsection

	Baseline Slow Gait Speed	Incident Slow Gait Speed	Normal Gait Speed at Baseline	Normal Gait Speed at 18 Months
ADAS-Cog (Baseline)	37.29 +/− 9.49	36.47 +/− 11.56	32.49 +/− 9.49	36.08 +/− 11.42
ADAS-Cog (18 Months)	44.70 +/− 16.01	47.28 +/− 15.91	41.53 +/− 14.43	40.96 +/− 14.22

### Baseline slow gait speed and incident falls over an 18 month duration

Overall, 57 (15.45%) participants experienced at least one fall over the 18 month duration of the trial. A small minority, 21 (5.69%), experienced recurrent (> 1) falls over the study period. Unadjusted, slow baseline gait speed was associated with increasing likelihood of falls (IRR: 3.18, 1.97–5.11, *p* < 0.001). Controlling for demographic and baseline AD variables, this association persisted (IRR: 3.27, 1.96–5.50, *p* < 0.001). Finally, after robust adjustment for important comorbidities and medications, the association remained significant (IRR: 3.48, 2.05–5.92, *p* < 0.001). See Table 6 for details. Under all models, slow gait speed was the strongest independent predictor of falls over the 18 month duration.

**Table 6:** Association between Baseline Slow Gait Speed and Falls at 18 Months in Mild to Moderate AD. Under all models, slow gait speed was significantly associated with falls (dependent variable) risk at 18 months. ADAS-Cog = Alzheimer Disease Assessment Scale – Cognitive Subsection

Predictor	Model 1		Model 2		Model 3	
	**IRR (95% CI)**	***P***	**IRR (95% CI)**	***P***	**IRR (95% CI)**	***P***
Baseline Slow Gait Speed	3.18 (1.97–5.11)	**< 0.001**	3.27 (1.96–5.50)	< 0.001*	3.09 (1.82–5.22)	< 0.001*
Baseline ADAS-Cog			0.95 (0.92–0.97)	0.001*	0.95 (0.92–0.98)	0.001*
Age			1.07 (1.04–1.10)	< 0.001*	1.07 (1.03–1.10)	< 0.001*
Gender (Female)			1.90 (1.15–3.11)	0.012*	1.60 (0.97–2.65)	0.068
Study Arm			1.20 (0.78–1.83)	0.409	1.32 (0.85–2.06)	0.218
Body Mass Index			0.98 (0.94, 1.03)	0.489	0.96 (0.92–1.01)	0.149
Education (Years)			1.07 (1.01–1.12)	0.021*	1.10 (1.04–1.15)	0.001*
Diagnosis Duration			1.02 (0.88–1.17)	0.816	0.96 (0.84–1.11)	0.612
Total Comorbidities					0.97 (0.88–1.07)	0.527
Diabetes Mellitus					1.73 (0.85–3.51)	0.128
Arthritis					2.19 (1.19–4.04)	0.012*
Total Medications					1.03 (0.95–1.12)	0.490
Benzodiazepine Use					1.05 (1.04–3.64)	0.036*
Antidepressant Use					0.67 (0.41–1.07)	0.096

## Discussion

In this study of older adults mild-to-moderate AD, greater cognitive impairment at baseline was significantly associated with both baseline slow gait speed and incident slow gait speed at 18 months. There was no association between baseline slow gait speed and longitudinal cognitive performance at 18 months. This is despite a clinically meaningful cognitive decline experienced by the cohort overall (with the mean ADAS-Cog score increasing by a mean of 8.87 points) [32]. Further, slow gait speed at baseline was associated with a more than threefold increased risk of falls over an 18 month period in those with mild to moderate AD. This is one of the first studies in the literature to assess the longitudinal relationship between slow gait speed and cognitive function in those with a diagnosis of AD

At baseline, the only significant predictors of slow gait speed were greater age and poorer cognition in multivariate analysis. This finding is noteworthy and is largely consistent with previous studies in the literature which have demonstrated a relationship between cognitive function and gait speed in a cross-sectional manner in those with AD [17–19].

The lack of association between baseline slow gait speed and cognitive outcomes is of interest. The only previous study in mild to moderate dementia to assess gait speed and cognition in a longitudinal fashion similarly found no association in longitudinal analysis for general cognition, but found a positive association for particular cognitive sub-domains, namely executive function [21]. One of the reasons for the lack of association in the current study may be that gait was considered as a dichotomous variable based on a pre-specified cut-off. It may be the case that more subtle abnormalities in gait speed may correlate with cognitive function and that such a stringent cut off may mask such an association. Nevertheless, it is possible that whilst slow gait speed may be a predictor of a later diagnosis of dementia, it may lose its predictive value once a diagnosis of dementia has been established.

One of the most interesting findings from the current analysis is the relationship between baseline cognitive function and incident slow gait speed over the duration of the study. Even in a fully adjusted model, baseline cognition was the strongest (apart from age) predictor of incident slow gait speed. This is particularly interesting given that 14% of the participants with normal gait speed at baseline converted to slow gait speed (incident slow gait speed). Thus, this may represent a subpopulation within those with mild-to-moderate AD who may be particularly vulnerable to adverse consequences.

The association between baseline slow gait speed and incident falls is particularly stark. Baseline slow gait speed was associated with a greater than threefold increased risk in incident falls over the 18 month study period. Indeed, baseline slow gait speed was the strongest predictor of incident falls under a fully adjusted model. This is particularly pertinent given that more than 15% of the participants with mild to moderate AD experienced at least one fall over the study period. Targeted interventions for falls prevention focussed on those with AD and slow gait speed are warranted and slow gait speed may be a way to select those with mild to moderate AD who are particularly vulnerable to experiencing falls, given the adverse consequences of falls in such a cohort [23].

An important consideration in the present study is the nature of the included cohort. Notably, patients with other neurological disorders such as Parkinson’s Disease were excluded. Further, patients with a significant cardiac history, previous stroke etc. were all excluded. Thus, the cohort of included patients had fewer comorbidities which may impair cognition than in other naturalistic studies. This enhances the current study in assessing the relationship between gait and cognition specifically in mild-to-moderate AD.

Other strengths of this study include its international nature as well as the high fidelity of follow up in included patients and the large amount of clinical and medical history data available. This enabled us to control for other causes of gait disorders such as arthritis and diabetes in our analysis. Further, it enabled us to include the effects of certain medications on incident slow gait speed and falls, such as benzodiazepines, which have been previously linked with falls in this vulnerable population [32].

Our study has several limitations. Principal amongst these is the fact that gait was assessed as a binary variable. Applying such a stringent cut-off in a dichotomous fashion may not produce the same results as analysis repeated with gait speed as a continuous variable in meters per second for instance. However, in the current study we were limited by the fact that gait speed was recorded as an individual binary variable and not a specific speed in metres per second. This may introduce less accuracy into our results than more detailed recording of gait speeds. Future longitudinal studies should examine the intricacies of the gait-cognition relationship in those with a diagnosis of dementia. A further limitation of the current study is that gait and cognition was only considered over an 18 month period. Such a period may be too short to demonstrate the relationship between gait speed and cognition. Previous studies in the literature have demonstrated a relationship over a longer period of time and we cannot rule out that following this cohort for a longer period of time may demonstrate a significant relationship between baseline gait speed and cognition at follow-up.

In conclusion, we demonstrate a significant baseline association between cognition and slow gait speed in a large cohort of participants with mild to moderate Alzheimer Disease. Poorer baseline cognition was associated with incident slow gait speed over 18 months. However, there was no association between baseline slow gait speed and cognition at follow-up. We also report a significant increase in falls risk over an 18 month period in those with baseline slow gait speed. Further studies should continue to explore the nature of the gait-cognition relationship specifically in those with Alzheimer Disease in helping to tease out which patients may be particularly vulnerable to further cognitive decline and adverse health outcomes.

## Data Availability

Due to the chance of individual participant identification in the current trial, data is not publicly available. Requests to obtain anonymised study data can be addressed to dyera@tcd.ie

## Abbreviations

AD: Alzheimer Disease
ADAS-Cog: Alzheimer’s Disease Assessment Scale, Cognitive Subsection
ATC: Anatomic Therapeutic Classification
BMI: Body Mass Index
CDR-Sb: Clinical Dementia Rating, Sum of Boxes
MCI: Mild Cognitive Impairment
NINCDS-ADRDA for AD: National Institute of Neurological and Communicative Disorders and Stroke/Alzheimer’s Disease Criteria
NILVAD: A European Multicentre Double Blind-Placebo Controlled Phase III Trial of Nilvadipine in Mild-to-Moderate Alzheimer’s Disease
